# *CBXs*-related prognostic gene signature correlates with immune microenvironment in gastric cancer

**DOI:** 10.18632/aging.204214

**Published:** 2022-08-14

**Authors:** Yin Jiang Zhang, Lin Yi Zhao, Xu He, Rong Fei Yao, Fan Lu, Bi Nan Lu, Zong Ran Pang

**Affiliations:** 1 School of Pharmacy, Minzu University of China, Beijing, P.R. China; 2 Key Laboratory of Ethnomedicine (Minzu University of China), Ministry of Education, Beijing, P.R. China

**Keywords:** chromobox proteins, gastric cancer, prognostic gene model, immune infiltration, risk score

## Abstract

Background: Chromobox (*CBX*) proteins are important Polycomb family proteins in the development of gastric cancer. Nonetheless, the relationship between *CBXs* and gastric cancer microenvironment remains unclear.

Methods: Multiple databases were used for the analysis of *CBXs* expression and clinical value in gastric cancer patients. A Cox regression analysis was used to evaluate the prognostic importance of *CBXs*. Thereafter, regression analysis of LASSO Cox was used to construct the prognostic model. Spearman's correlation between risk score and immune infiltration was analyzed using the McP-counter algorithm. A predicted nomogram was developed to predict the overall survival of gastric cancer patients after 1, 2, and 3 years.

Results: In contrast with normal tissues, mRNA and protein expression levels of *CBX2/3* were significantly high in gastric cancer tissues, whereas those of *CBX6/7* were low. *CBXs* significantly correlated with immune subtypes and molecular subtypes. A prognostic gene model based on five *CBX* genes (*CBX1*, *CBX2*, *CBX3*, *CBX7,* and *CBX8*) predicted the overall survival of gastric cancer patients. A significant correlation was noted between the risk score of the CBXs-related prognostic gene model and immune-cell infiltration. Low risk patients could achieve a better response to immune checkpoint inhibitors. A predictive nomogram constructed using the above five *CBX* genes revealed that overall survival rates over 1, 2, and 3 years could be reasonably predicted. Therefore, the roles of *CBXs* were associated with chromatin modifications and histone methylation, etc.

Conclusion: In summary, we identified a prognostic *CBXs* model comprising five genes (*CBX1*, *CBX2*, *CBX3*, *CBX7,* and *CBX8*) for gastric cancer patients through bioinformatics analysis.

## INTRODUCTION

Gastric cancer is the 4th most common type of cancer, with a global incidence of approximately 1 million annually [1]. Many risk factors are implicated in the etiology of gastric cancer, including *Helicobacter pylori* infection, lack of fiber food, irregular food intake, heredity, etc. [2]. Treatment options for gastric cancer are limited [3]. Many studies have explored the mechanisms involved in the development, progression, and metastases of gastric cancer. Nevertheless, the molecular mechanism of gastric cancer in tumor microenvironments remains unclear. Therefore, unraveling the pathogenesis of gastric cancer in the tumor microenvironment will facilitate the identification of diagnostic biomarkers and the development of novel treatment strategies [4].

Tumor microenvironments comprise heterogeneous populations, including gastric cancer cells and infiltrating immune cells, which are essential regulators of cancer development. A single-cell RNA sequencing of TME revealed the immune cell landscape at the single-cell level, which helps in identifying novel clusters of tumor-associated immune cells [5] and signature genes for different immune cells. In gastric cancer tissues, a down-regulated *IRF8* transcription factor was reported in CD8+ tumor-infiltrating lymphocytes [6]. Pembrolizumab, an immune checkpoint inhibitor that targets *PD-1* and its *PD-1* interactions with *PD-L1* and *PD-L2*, is a therapeutic approach for gastric cancer [7, 8]. Stratifying patients based on molecular and genomic signatures is essential to identify suitable immunotherapeutic methods for each subgroup.

Polycomb group (*PcG*) proteins are essential gene regulators that mediate the stable inheritance of cell states. Aberration of epigenetic regulation mediated by *PcG* proteins has been explored in several cancer types. The *CBX* protein family, critical canonical *PcG* components, regulate tumorigenesis and tumor progression by maintaining tumor suppressors and the undifferentiated state of cancer stem cells [9]. Eight members of *CBX* proteins have been identified in human genomes. These *CBXs* regulate heterochromatin, mediation of *PRC1* binding to nucleosomes, recruitment as well as stabilization of *PRC1* to distinct chromatin regions. These proteins have a conserved N-terminal chromodomain. Two groups of *CBXs* have been defined based on differences in molecular structures and functions. The heterochromatin protein *1β* (*HP1β*) group contains *CBX1/3/5*, which are associated with the heterochromatin protein 1 (*HP1*) complex to interpret *H3K9me3* marks mediated by *H3K9* methyltransferases. The Pc group has a conserved C-terminal polycomb repressor box, comprising *CBX2/4/6/7/8*, deposited by polycomb repressive complex 2 to recognize *H3K27me3* [10]. Previous studies have shown the aberrant expressions of *CBX* family proteins and their prognostic values in gastric cancer [11, 12]. For instance, *CBX6* is up-regulated in hepatocellular carcinoma and associated with lower survival outcomes [13]. *CBX7* positively regulates the phenotype of gastric cancer stem cells by downregulating *p16* and upregulating *microRNA-21* [14]. Nevertheless, the correlation between *CBXs* and immune cell infiltration in the gastric cancer microenvironment remains elusive.

This study investigated the expression levels, clinical stages, mutations, risk factors, copy number variations (CNVs), and the immune microenvironment of gastric cancer. Consequently, we found that a prognostic *CBXs* model containing five *CBX* genes could predict overall survival for gastric cancer patients. Besides, a significant correlation was noted between the risk score of the *CBXs*-related prognostic gene model and immune-cell infiltration.

## RESULTS

### Expression levels of different *CBXs* family members

First, we determined the expression levels of *CBXs* in different cancer types using the ONCOMINE database. Significantly upregulated mRNA expression of *CBX1/2/3/4* was discovered in gastric cancer tissues compared to in normal control tissues (Supplementary Figure 1). Among the 8 *CBXs* family members, the expression levels of *CBX1/3* were significantly upregulated, whereas *CBX7* expression was significantly downregulated in other cancer types. Supplementary Table 1 summarizes the studies on gastric cancer. *CBX1/2/3/4/6* were significantly up-regulated in different gastric adenocarcinoma types, whereas *CBX7* was significantly down-regulated in diffuse gastric adenocarcinoma. These findings are in line with observation in different cancer types, which indicates the conserved function of the *CBXs* family among various tumor types.

**Figure 1:**
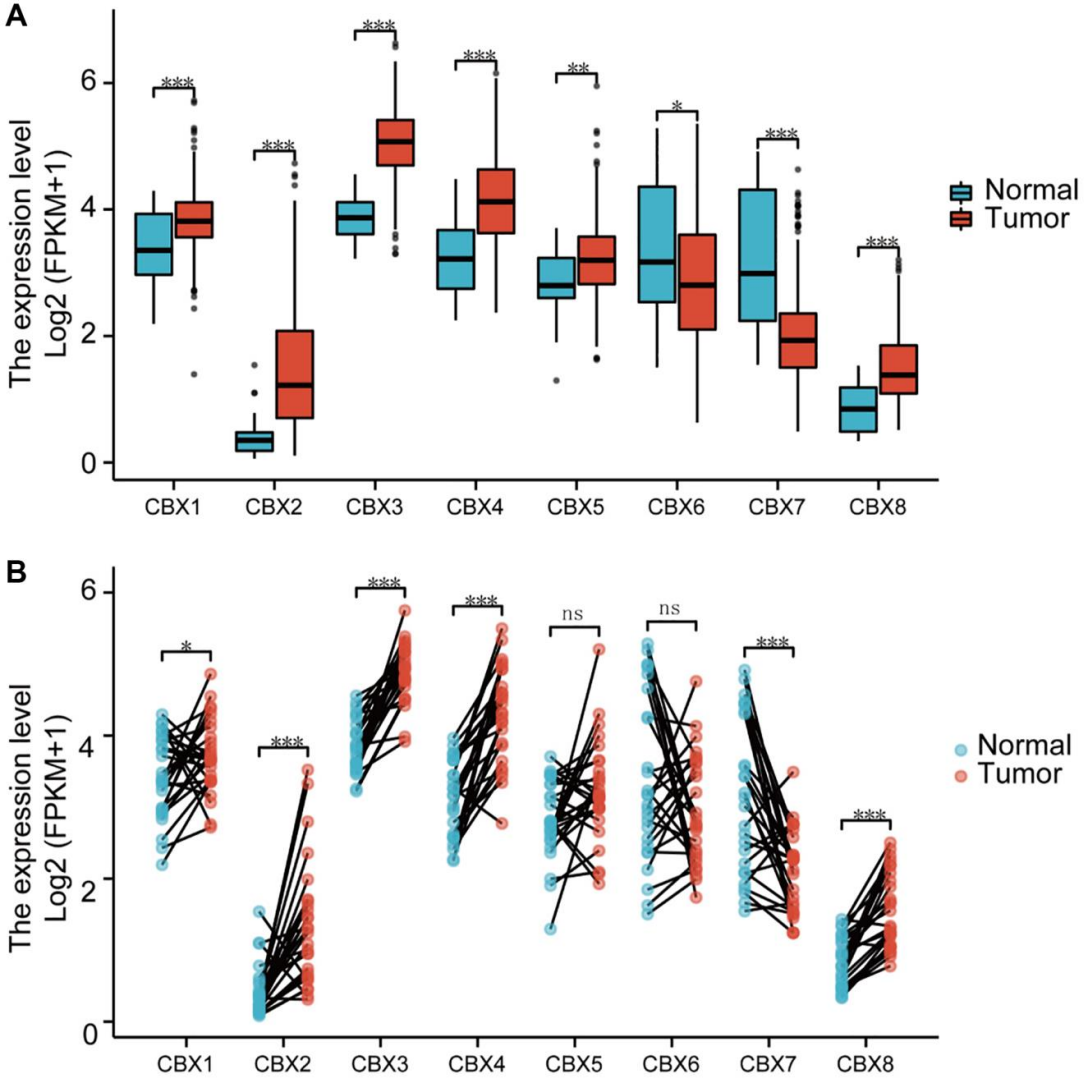
**Analysis of *CBXs* mRNA expression levels.** (**A**) unpaired samples containing 32 normal tissues and 375 gastric cancer tissues; (**B**) paired samples containing 32 normal tissues and corresponding gastric cancer tissues. Wilcoxon rank-sum test was used. ^*^p < 0.05; ^**^p < 0.01; ^***^p < 0.001; Abbreviation: ns: not significant.

RNA-seq data were downloaded from the TCGA, including 32 normal tissues and 375 gastric cancer tissues to verify the mRNA expression patterns of 8 *CBXs* in gastric cancer. Expression levels of *CBX1/2/3/4/8* in gastric cancer samples were significantly upregulated, whereas mRNA expressions of *CBX7* were significantly downregulated compared to the normal control in unpaired and paired analysis (Figure 1). The mRNA expression levels of 408 gastric cancer tissues were compared with 211 normal tissues using GEPIA online database. As shown in Supplementary Figure 2, the mRNA levels of *CBX1/2/3/5/8* in gastric cancer tissues were significantly upregulated. *CBX7* expression was significantly down-regulated in tumor samples, consistent with outcomes in other gastric cancer types (Supplementary Table 1).

Further, we evaluated protein expression patterns of *CBXs* in gastric cancer (Supplementary Figure 3). Protein levels of *CBX2/3* increased in gastric cancer tissues. Suppressed protein expressions of *CBX4/6/7* were observed in gastric cancer tissues. Additionally, similar protein expression levels of *CBX5/8* were observed between normal tissues and gastric cancer tissues. Protein expression levels of *CBX2/3/6/7* were in line with changes in mRNA expression levels.

### Relationship between *CBXs* and clinicopathological features of patients with gastric cancer

We investigated the relationship between mRNA expression of *CBXs* and the clinical stage of gastric cancer patients. The mRNA expression levels of *CBX*s were not correlated with tumor stages in both databases (Supplementary Figures 4 and 5).

The relationship between *CBXs* mRNA expression levels and gastric cancer clinical grades was evaluated using the TISIDB database. The mRNA expression levels appeared high in patients with advanced cancer grades. Expression levels of *CBX3/4/6/7/8* were significantly upregulated with clinical grades (Supplementary Figure 6). The highest mRNA expressions of *CBX3/4/8* were found in grade 2, and the expression level dropped from grade 2 to 3 as the tumor grade increased. The highest mRNA expressions of CBX6/7 were found in grade 3. However, the expression levels of *CBX1/2/5* did not significantly change with clinical grade. By integrating the results of mRNA and protein expression levels, *CBX3/4/8* expression levels increased significantly from clinical grade 1 to 2 in gastric cancer.

### Prognostic value of *CBXs* in gastric cancer patients

The correlation between *CBXs* and clinical outcomes in gastric cancer patients was examined using the microarray dataset and the RNA-seq data. The microarray dataset revealed that upregulated mRNA expressions of *CBX3* (HR = 0.59, *P* = 1.4E-09) caused better overall survival outcomes among gastric cancer patients. In comparison, upregulated mRNA expression levels of *CBX4* (HR = 1.25, *p* = 0.041), *CBX*5 (HR=2.08, *p* = 1.3E-13), *CBX6* (HR = 1.5, *p* = 3.4E-06), *CBX7* (HR = 1.52, *p* = 2e-06), and *CBX8* (HR = 2.36, *p* = 3.1E-14) correlated with poor overall survival outcomes (Supplementary Figure 7). In addition, the RNA-seq data revealed that the mRNA expression levels of *CBX1* (HR=1.61, *p* = 0.02) and *CBX8* (HR = 0.62, *p* = 0.0048) significantly correlated with clinical outcomes in gastric cancer (Supplementary Figure 8). In general, mRNA expression levels of *CBX1/3/4/5/6/7/8* significantly contributed to gastric cancer prognosis, confirming their potential application as biomarkers for the prediction of survival outcomes in gastric cancer patients.

### Immune cell infiltration of *CBXs* in gastric cancer patients

**Figure 2:**
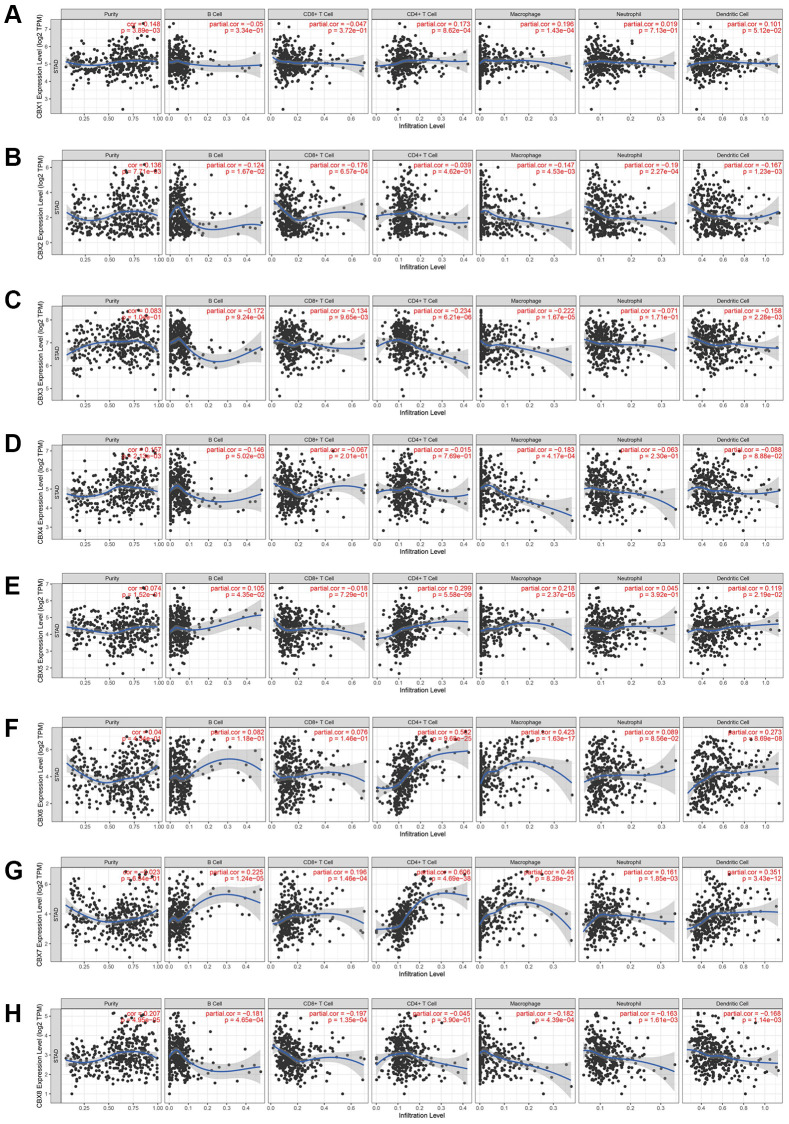
**The correlation between *CBXs* and immune cell infiltration was analyzed by the TIMER database.** (**A**) *CBX1*; (**B**) *CBX2*; (**C**) *CBX3*; (**D**) *CBX4*; (**E**) *CBX5*; (**F**) *CBX6*; (**G**) *CBX7*; (**H**) *CBX8*.

A positive correlation was noted between *CBX1* expression and *CD4+ T* cells as well as macrophage infiltration (Figure 2A). *CBX2* and *CBX8* expression levels inhibited CD8+ T cells, macrophages, neutrophils, and dendritic cells infiltration (Figure 2B, 2H). A negative correlation was noted between *CBX3* expression and B cells, CD8+ T cells, CD4+ T cells, macrophages, neutrophils, and dendritic cell infiltration (Figure 2C). *CBX4* expression levels promoted B cell infiltration but suppressed macrophage infiltration (Figure 2D). Moreover, *CBX5* expression promoted CD4+ T cells and macrophage infiltration (Figure 2E). A negative correlation was noted between *CBX6* expression and CD4+ T cells, macrophages, and dendritic cell infiltration (Figure 2F). *CBX7* expression promoted all types of immune cell infiltration (Figure 2G).

If a correlation coefficient >0.3 was defined as a strong correlation, then *CBX6* promoted infiltration of CD4+ T cells (*P* = 9.28e-25, Cor = 0.502) and macrophages (*P* = 1.63e-17, Cor = 0.423), whereas *CBX7* promoted the infiltration of CD4+ T cells (*P* = 4.69e-38, Cor = 0.606), macrophages (*P* = 8.28e-21, Cor = 0.46), and dendritic cells (*P* = 3.43e-12, Cor = 0.351).

Moreover, correlations between *CBXs* expression and tumor-infiltrating lymphocytes were discovered in various cancer types (Supplementary Figure 9).

**Figure 3:**
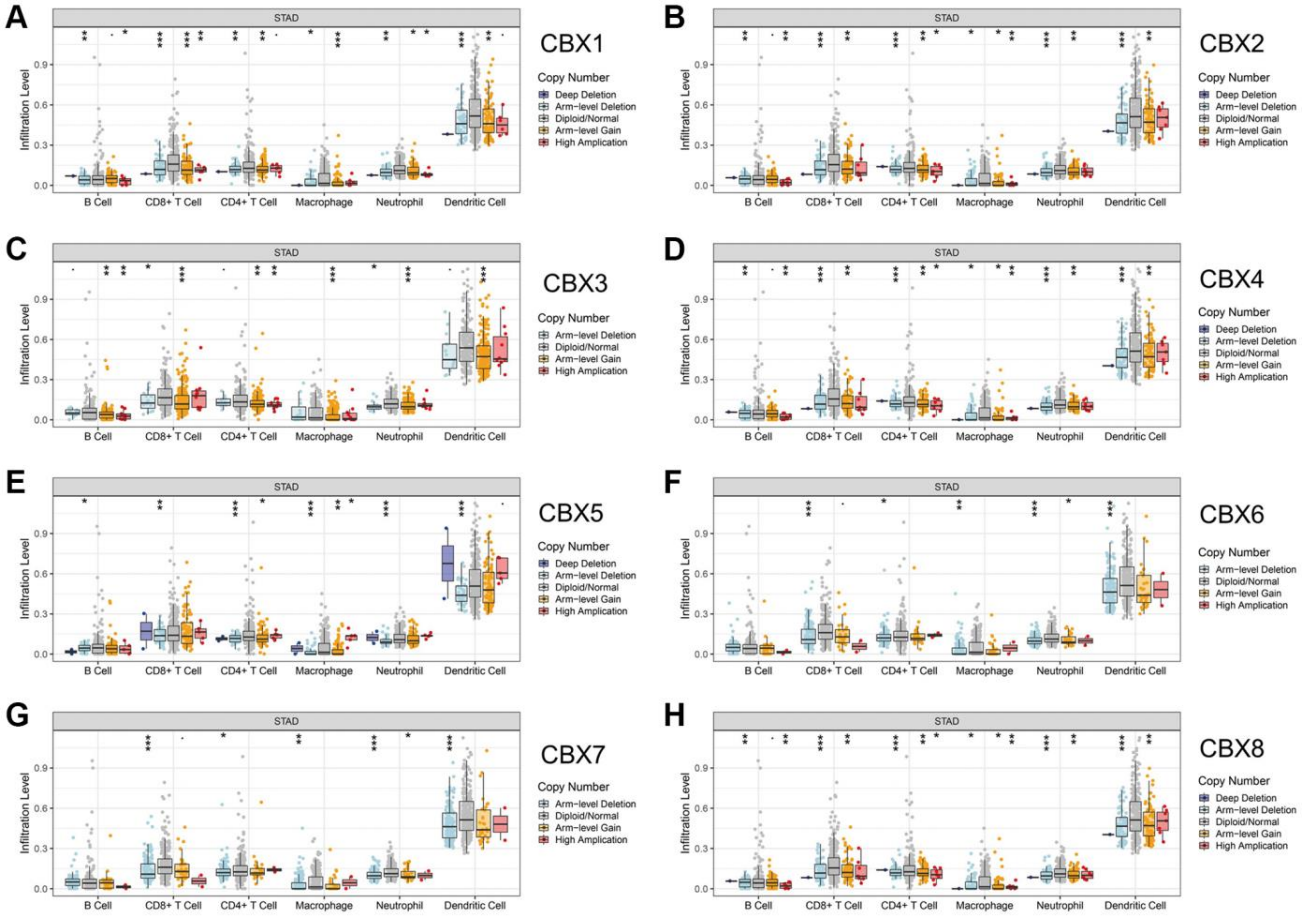
**Correlation between CNV of *CBXs* and immune cell infiltration in gastric cancer analyzed by TIMER.** (**A**) *CBX1*; (**B**) *CBX2*; (**C**) *CBX3*; (**D**) *CBX4*; (**E**) *CBX5*; (**F**) *CBX6*; (**G**) *CBX7*; (**H**) *CBX8*. ^*^p < 0.05; ^**^p < 0.01; ^***^p < 0.001.

The relationship between CNVs of *CBXs* and immune cell infiltration was evaluated. The CNVs of *CBXs* significantly correlated with immune cells, including B cells, CD8+ T cells, CD4+ T cells, macrophages, neutrophils, and dendritic cell infiltrations (Figure 3).

### Relationship between mRNA expressions of *CBXs* with immune subtypes and molecular subtypes in gastric cancer patients

**Figure 4:**
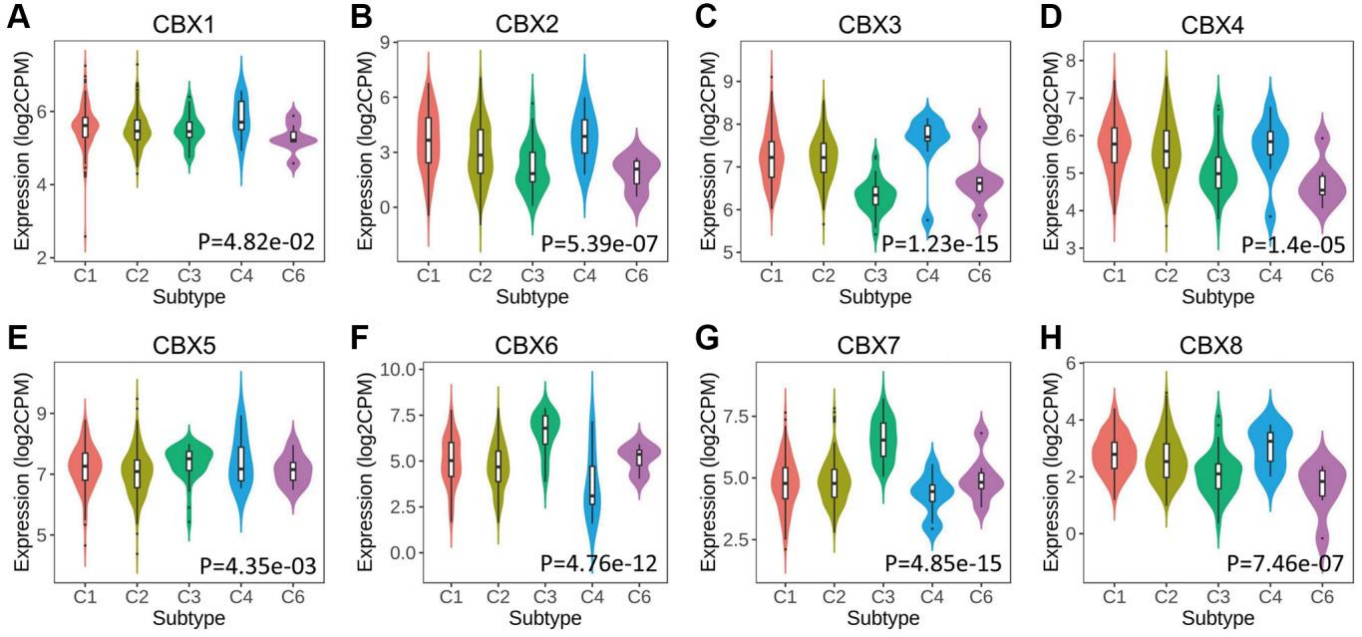
**Relationship between *CBXs* and immune subtypes across in gastric cancer in TISIDB.** (**A**) *CBX1*; (**B**) *CBX2*; (**C**) *CBX3*; (**D**) *CBX4*; (**E**) *CBX5*; (**F**) *CBX6*; (**G**) *CBX7*; (**H**) *CBX8*. C1: wound healing (*n* = 129); C2: *IFN*-gamma dominant (*n* = 210); C3: inflammatory (*n* = 36); C4: lymphocyte depleted (*n* = 9); C6: *TGF-β* dominant (*n* = 7).

*CBXs* significantly correlated with five immune subtypes analyzed in the TISIDB database (Figure 4). Expression levels of *CBX1/2/3/4/8* in lymphocyte depleted subtype (C4) were significantly greater than those in other subtypes, and *CBX6/7* had a significantly lower expression level in C4 and a higher expression level in inflammatory subtype (C3).

**Figure 5:**
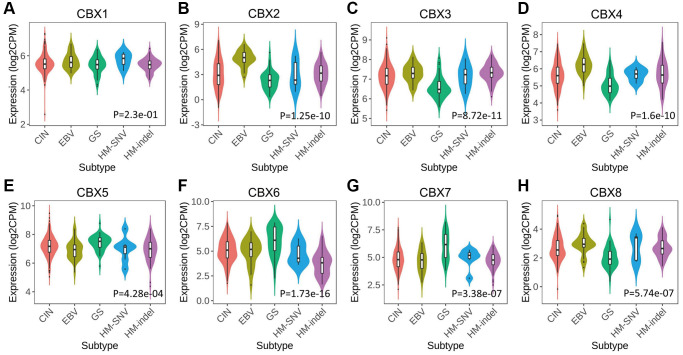
**Relationship between *CBXs* and molecular subtypes in gastric cancer in TISIDB.** (**A**) *CBX1*; (**B**) *CBX2*; (**C**) *CBX3*; (**D**) *CBX4*; (**E**) *CBX5*; (**F**) *CBX6*; (**G**) *CBX7*; (**H**) *CBX8*. Abbreviations: CIN: chromosomal instability (*n* = 223); EBV: Epstein–Barr virus positive (*n* = 30); GS: genomically stable (*n* = 50); HM-SNV: hypermutated-single-nucleotide variant predominant (*n* = 7) and HM-indel: hypermutated-insertion deletion mutation (*n* = 73).

The relationships between *CBXs* and the five molecular subtypes were investigated in the TISIDB database. Expression levels of *CBXs* except for *CBX1* significantly correlated with five molecular subtypes. Expression levels of *CBX2/3/4/8* in the genomically stable (GS) subtype were significantly low compared to the other subtypes, and *CBX5/6/7* exhibited a higher expression level in the GS subtype (Figure 5).

### Relationship between *CBXs*-related gene model and tumor immune infiltration

**Figure 6:**
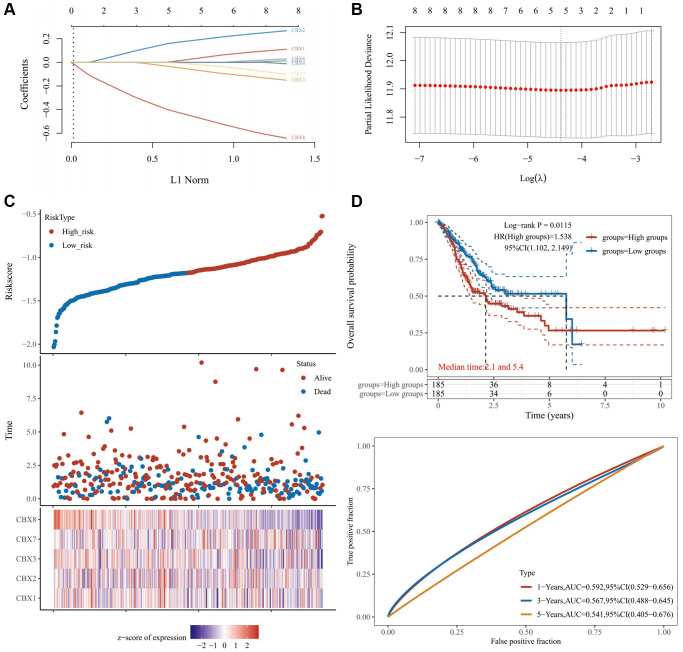
**Constructing a prognosis model of *CBXs*.** (**A**) LASSO index profiles of the five *CBXs*; (**B**) Plots of the ten-fold cross-validation error rates; (**C**) The distribution of risk score, survival status, and the expression of 5 genes in gastric cancer; (**D**) Overall survival curves for gastric cancer patients in the high-/low-risk group and the ROC curve of measuring the predictive value. Abbreviation: *CBXs*, chromobox proteins.

**Figure 7:**
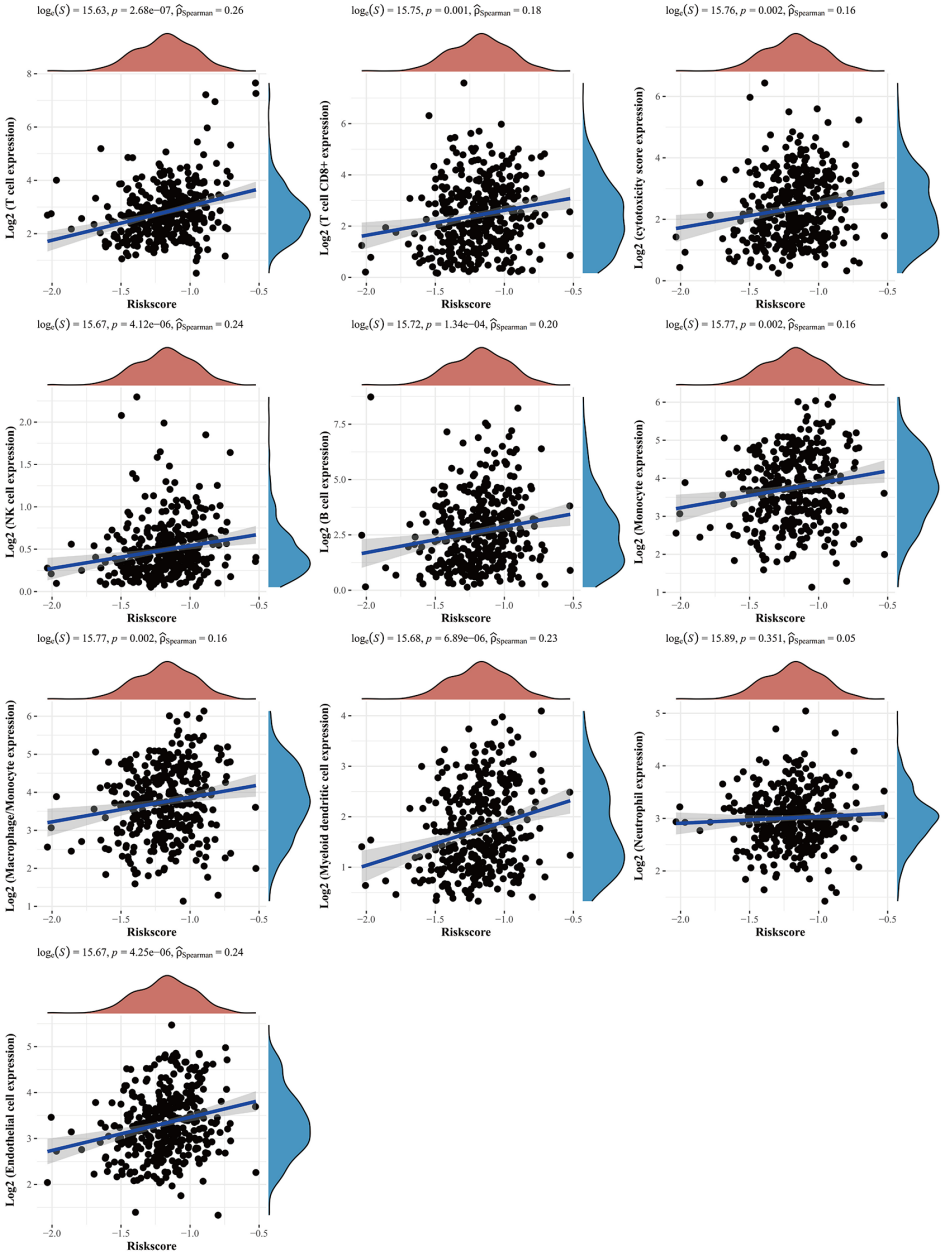
**The relationship between risk score and immune infiltration.** The relationship between the abundance of immune cells and the risk score of prognostic *CBXs* model in gastric cancer. Abbreviation: *CBXs*, chromobox proteins.

The regression analysis of LASSO Cox was performed to construct a prognostic gene model based on these five prognostic *CBXs* (Figure 6A, 6B). Risk score = (0.0327) × *CBX1* + (0.1882) × *CBX2* + (−0.0651) × *CBX3* + (−0.0178) × *CBX7* + (−0.4636) × *CBX8*. Based on this risk score, gastric cancer patients were categorized into two groups. Figure 6C shows the distribution of risk score, survival status, and expression of these 5 genes. With an increased risk score, the risk of patient death increased, whereas the survival time decreased (Figure 6C). Kaplan-Meier curves revealed a lower overall survival probability of gastric cancer patients with a high-risk score than those with a low-risk score (median time = 2.1 years vs. 5.4 years, *p* = 0.0115, Figure 6D), with AUCs of 0.592, 0.567, and 0.541 in ROC curves at 1, 3 and 5 years, respectively. Meanwhile, we also found that the overall survival probability of gastric cancer patients with a high risk score was lower than that of patients with a low risk score in GSE84437 data sets (*p* = 0.016, Supplementary Figure 10), with AUCs of 0.469, 0.530, and 0.543 in ROC curves at 1, 3 and 5 years, respectively. Thereafter, the relationship between the risk score of the *CBXs*-related prognostic model and immune cell infiltration was evaluated using the MCP-counter method. Consequently, we found a significant positive correlation between the risk score and immune cell infiltration (Figure 7, *p* < 0.05). Furthermore, there were significant differences in immune checkpoints between low and high risk patients in TCGA-STAD (Supplementary Figure 11A) and GSE84437 (Supplementary Figure 11B) data sets.

**Figure 8:**
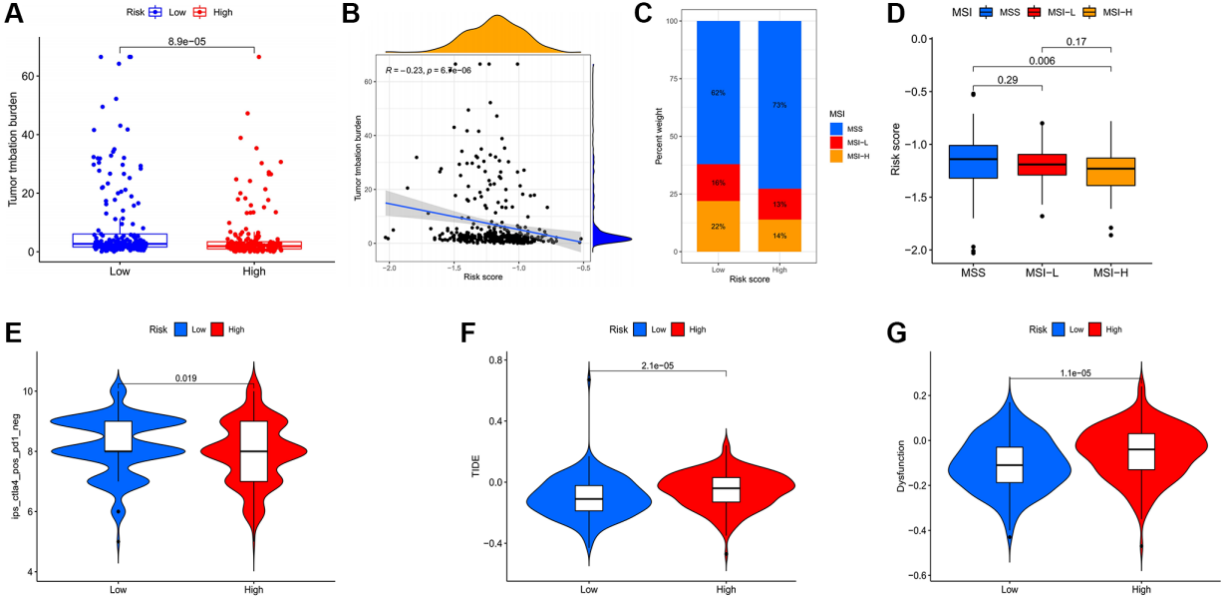
**Evaluation of the correlation between risk scores and potential response to immunotherapy for gastric cancer patients.** (**A**) Higher TMB in the low risk group. (**B**) TMB was negatively correlated with risk score. (**C**) Associations between the risk score and MSI. (**D**) MSI-H in the low risk group. (**E**) The association between the risk score and the Immunophenoscore (IPS) of anti-CTLA4 monotherapy. Higher TIDE (**F**) and T cell dysfunction (**G**) score in the high risk group.

In addition to well-known TMB [15] and MSI [16], newly identified predictors, such as IPS [17] and TIDE [18], are widely used to evaluate the immune response. Our analysis revealed that the low risk group had higher mutation frequencies (Figure 8A) and the risk score and TMB also exhibited a significant negative correlation (Figure 8B). The low risk group had higher MSI (Figure 8C, 8D). The IPS was significantly elevated in the low risk group (Figure 8E). TIDE and T cell dysfunction were significantly decreased in the low risk group (Figure 8F, 8G). These findings demonstrated that low risk patients would achieve a better response to immune checkpoint inhibitors.

### Construction of a predictive nomogram

**Figure 9:**
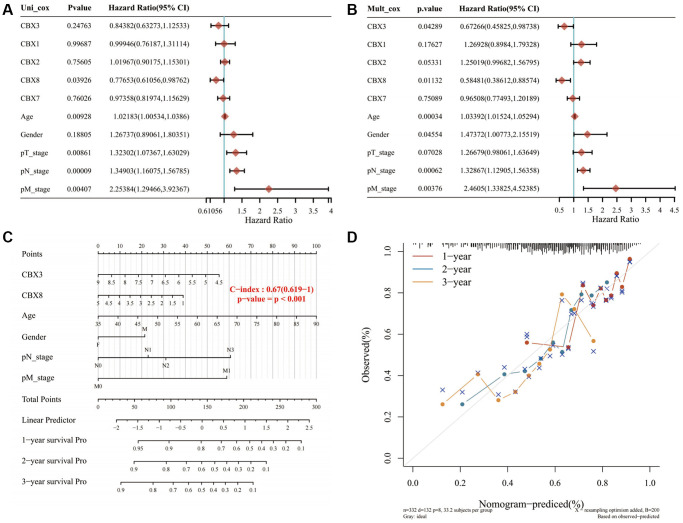
**Constructing a prediction nomogram.** (**A**, **B**) Univariate and multivariate regression analysis showed that *CBX3*, *CBX8*, age, gender, pT stage, pN stage, and pM stage were independent factors for the prognosis of gastric cancer patients; (**C**, **D**). The predictive nomogram suggested that overall survival rates over 1, 2 and 3 years could be reasonably predicted. A dashed diagonal line represents the ideal nomogram. Abbreviation: *CBXs*, chromobox proteins.

Univariate and multivariate regression analyses revealed that *CBX3*, *CBX8*, age, gender, pT stage, pN stage, and pM stage were independent factors for the prognosis of gastric cancer patients (Figure 9A, 9B). The predictive nomogram revealed that overall survival rates over 1, 2, and 3 years could be reasonably predicted (Figure 9C, 9D).

### Risk factors associated with gastric cancer mortality

Further, the risk factors associated with mortality in 359 gastric cancer patients were assessed, among whom 140 died in the TIMER database. Table 1 presents a Cox proportional hazard model used to evaluate risk factors for mortality. Multivariate analysis revealed that six variables were risk factors for mortality in gastric cancer: stage II (HR = 2.125, *p* = 0.04); stage III (HR = 3.223, *p* = 0.001); stage IV (HR = 7.01, *p* < 0.001); age (HR = 1.042, *p* < 0.001); macrophages (HR = 475.661, *p* = 0.001), and *CBX8* (HR = 0.595, *p* = 0.042) these variables were significantly associated with clinical outcomes of gastric cancer patients (Table 1). *CBX6/7* significantly correlated with CD4+ T cells and macrophages, hence might be promising risk factors.

**Table 1:** The Cox regression model of clinical factors, tumor-infiltrating immune cells, and *CBXs* were analyzed by the TIMER database.

	**coef**	**HR**	**95% CI_low**	**95% CI_up**	***P* value**	**sig**
Stage II	0.754	2.125	1.034	4.365	0.04	^*^
Stage III	1.17	3.223	1.66	6.258	0.001	^**^
Stage IV	1.947	7.01	3.247	15.137	0	^***^
Age	0.041	1.042	1.023	1.061	0	^***^
Gender (male)	0.193	1.213	0.832	1.768	0.315	ns
B cell	3.082	21.802	0.244	1948.612	0.179	ns
CD8_Tcell	−1.731	0.177	0.008	3.94	0.274	ns
CD4_Tcell	−1.64	0.194	0.001	37.011	0.54	ns
Macrophage	6.165	475.661	11.501	19672.08	0.001	^**^
Neutrophil	−3.88	0.021	0	7.893	0.201	ns
Dendritic	1.25	3.491	0.267	45.573	0.34	ns
*CBX1*	0.25	1.284	0.9	1.833	0.168	ns
*CBX2*	0.137	1.146	0.904	1.453	0.258	ns
*CBX3*	−0.166	0.847	0.57	1.257	0.41	ns
*CBX4*	0.126	1.134	0.759	1.695	0.538	ns
*CBX5*	0.06	1.062	0.791	1.426	0.69	ns
*CBX6*	0.068	1.07	0.863	1.328	0.537	ns
*CBX7*	−0.246	0.782	0.591	1.035	0.086	ns
*CBX8*	−0.52	0.595	0.36	0.982	0.042	^*^

Abbreviations: Coef: a regression coefficient; HR: hazard ratio; 95% CI: 95% confidential interval. ^*^p < 0.05; ^**^p < 0.01; ^***^p < 0.001; ns, not significant.

**Figure 10:**
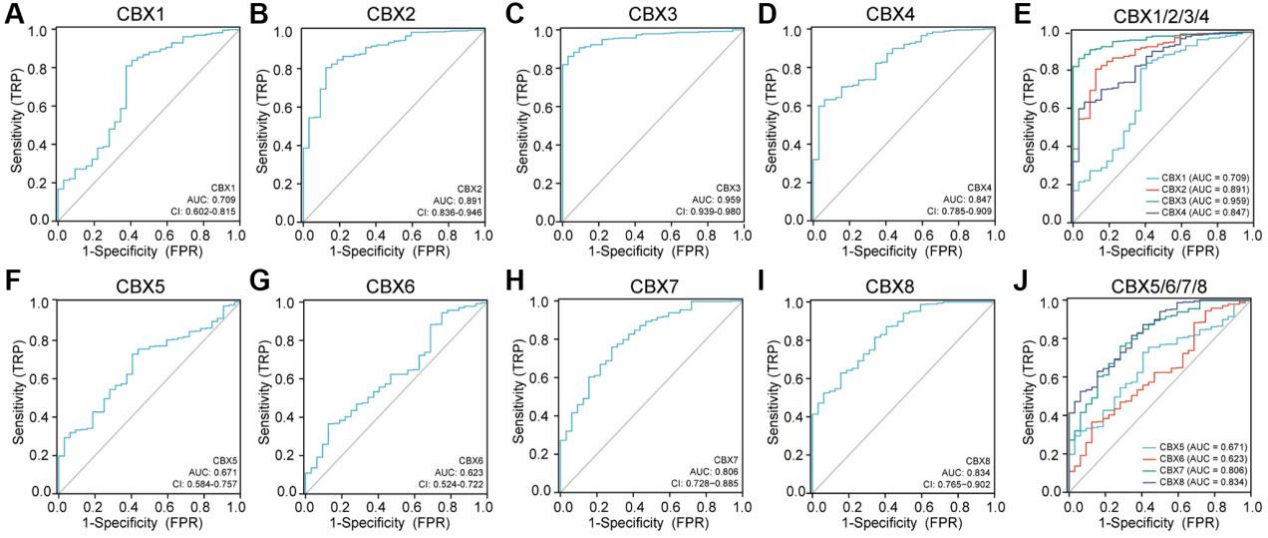
**Receiver operating characteristic (ROC) curves for each *CBXs* gene in gastric cancer.** (**A**) *CBX1*; (**B**) *CBX2*; (**C**) *CBX3*; (**D**) *CBX4*; (**E**) *CBX5*; (**F**) *CBX6*; (**G**) *CBX7*; (**H**) *CBX8*; (**I**) *CBX1/2/3/4*; (**J**) *CBX5/6/7/8*. Abbreviations: CI: confidence interval; AUC: area under curve; FPR: false positive rate; TPR: true positive rate.

Figure 10 shows the receiver operating characteristic (ROC) curves for each *CBXs* gene. The area under the curve (AUC) for *CBX3* was the highest at 0.959, indicating that *CBX3*-based prognostic indicators exert the best effect on patient stratification. Additionally, the AUC for *CBX2/4/7/8* was more than 0.8, indicating the predictive efficacy of these genes.

### Functional enrichment analysis of *CBXs*

**Figure 11:**
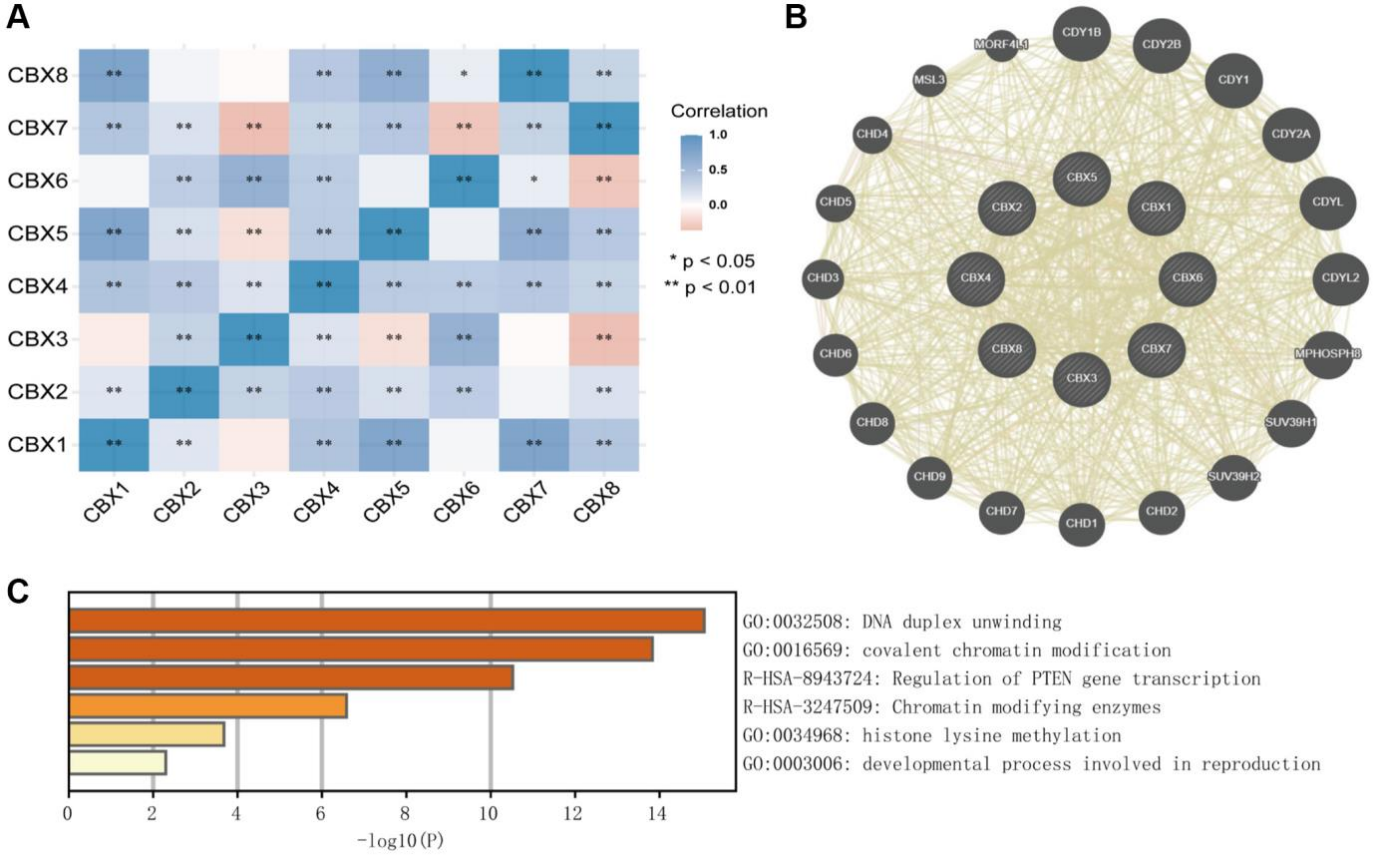
Predicted functions and pathways of *CBXs* and their coexpression neighbor genes in gastric cancer by Correlation heatmap (**A**), GeneMANIA (**B**), and Metascape (**C**).

A moderate to high correlation was observed in *CBX2*, *CBX3*, *CBX4*, *CBX6*, and *CBX7*, and a high correlation among *CBX1*, *CBX5*, and *CBX8* (Figure 11A). Co-expression neighbor gene analysis of differentially expressed *CBXs* was performed using the GeneMANIA to explore potential interactions among them (Figure 11B). Metascape was used to analyze the functions of *CBXs* and their neighboring genes. As a result, GO term and pathways, including DNA duplex unwinding, covalent chromatin modification, regulation of PTEN gene transcription, chromatin-modifying enzymes, histone lysine methylation, and developmental processes involved in reproduction were linked to *CBXs* functions in gastric cancer (Figure 11C).

### Drug targets, miRNA targets, and transcription factor targets of *CBXs*

Drug targets, miRNA, and transcription factor targets of *CBXs* were investigated using the Enrichr databases. Three drugs, including Prednisolone, Phenacetin, and Pramoxine were identified for targeting *CBXs* in gastric cancer (Supplementary Table 2). The top three miRNA targets of *CBXs* included mmu-miR-493, his-miR-1296, and mmu-miR-5128 (Supplementary Table 3). Besides, the top three transcription factors (*TEAD4*, *NRF1,* and *HINFP*) were associated with the regulation of *CBXs* (Supplementary Table 4).

## DISCUSSION

Dysregulation of *CBX* family proteins has been analyzed in various cancer types [9, 19–21]. Evidence suggests that *CBXs* regulate tumorigenesis, tumor cell proliferation, invasion, and metastasis [10, 22]. Research has identified a correlation between *CBX* proteins and the tumor microenvironment [23]. Nonetheless, the tumorigenesis role of the *CBXs* family, specifically intercellular communication with immune cell infiltration remains understudied. Herein, we comprehensively analyzed 8 *CBXs* in gastric cancer as per their expression patterns, protein expression levels, clinicopathological parameters, prognostic values, biological functions, immune cell infiltration, copy number variation, and ROC curve of *CBXs*.

The mRNA and protein expression levels of *CBX2/3* in gastric cancer tissues were significantly higher than in normal tissues, whereas *CBX6/7* were down-regulated in gastric cancer. Protein expression levels of *CBX1/2/5/8* were inconsistent with mRNA expression levels due to post-translational modification of *CBX* proteins. Studies have documented phosphorylation, SUMOylation/de-SUMOylation, and methylation/demethylation of *CBXs*. *CBX4* is also a *SUMO E3* ligase implicated in the regulation of SUMOylation and de-SUMOylation, and SUMOylation, mediating *PRC1* recruitment of methylated histone 3 at *K27* (*H3K27me3*), resulting in transcriptional repression of *Gata4/6* transcription [24].

Furthermore, we noted that mRNA expression levels of *CBX1/3/4/5/6/7/8* were significantly associated with gastric cancer prognosis. Notably, tumor stage and grade progression are influenced by protein expression levels, genetic mutations, tumor microenvironment, etc. We also observed dysregulated transcriptional expression of *CBXs* as tumors progressed. Studies indicate that *CBX1* overexpression in gastric and breast cancers significantly correlates with shorter overall survival outcomes. Evidence shows that interactions between tumor and immune cells modulate tumor progression and recurrence, and consequently immunotherapeutic responses as well as clinical outcomes. *CBX6/7* significantly correlates with immune cell infiltrations, particularly CD4+ T cells and macrophages, indicating that *CBXs* may also reveal immune status, hence regulating tumor status. We also revealed that the CNV of *CBXs* significantly correlates with immune cells. These results imply that *CBXs* could be critical regulators in gastric cancer progression. Previous studies have shown that *CBX7*-deficient upregulates *FasL* expression and consequently regulates CD4+ T cell apoptosis [25]. *CBX2* promotes virus-infected macrophages by improving *IFN-β* transcription and promoting *Jmjd3* recruitment to the *Ifnb* promoter [26]. The effects of *CBX* proteins on tumor states by regulating immune cell infiltrations warrant additional research. In addition, there were significant differences in immune checkpoints between low and high risk patients. The low risk patients could achieve a better response to immune checkpoint inhibitors.

Functional characterization of these genes revealed their relationship with chromatin modification and histone methylation. This was consistent with the roles of *CBXs*, a component of epigenetic regulation mediating proteins, PcG. We evaluated drug targets, miRNA targets, and transcription factor targets of the differentially expressed *CBXs*, and discovered that *TEAD4*, *NRF1*, and *HINFP* are critical transcription factors in the regulation of *CBXs*. Notably, *TEAD4* is a downstream effector of the Hippo pathway. In coordination with *YAP*, *TAZ*, and *VGLL*, *TEAD4* plays a critical role in cancer proliferation, including cell proliferation, metastasis, and cancer stem cell maintenance [27]. *NRF1* mediates drug resistance in cancer via an oncometabolite, *UDP-GlcNAc*, which stimulates proteasome subunit genes in response to proteasome inhibitors, before maintaining proteasome activity and protecting cancer cells from proteotoxicity [28]. *HINPF* ablation inhibits histone *H4* expression, disrupts the sub-nuclear organization of Histone Locus Bodies, and generates chromosomal fragility, hence sensitizing DNA to damage [29].

## CONCLUSION

In conclusion, we comprehensively analyzed the potential effects of *CBX* protein family members in gastric cancer. Consequently, *CBXs* correlated with overall survival outcomes and could be vital prognostic markers in gastric cancer. Moreover, we found a prognostic *CBXs* model comprising five genes (*CBX1*, *CBX2*, *CBX3*, *CBX7,* and *CBX8*) for gastric cancer patients. Additional experiments and clinical cohort studies for *CBXs* are necessary to validate our results further.

## MATERIALS AND METHODS

### ONCOMINE database

The ONCOMINE database (http://www.oncomine.org/) allows genome-wide expression analysis of integrated cancer microarray data [30]. Transcriptional expression of *CBXs* was investigated in gastric cancer tissues. Statistical differences in transcriptional expression levels between normal and cancer tissues were analyzed using the student’s *t-*test. Threshold settings were: *P*-value: 0.01; fold change: 1.5; gene rank: 10%; data type: mRNA.

### The cancer genome atlas database

The cancer genome atlas (TCGA, https://www.cancer.gov/tcga) is a landmark cancer genome project, comprising sequencing and pathological data of 33 cancer types [31]. We downloaded HTSeq-FPKM formatted RNA-seq data, corresponding clinical data, and somatic mutation information of gastric cancer, including 375 tumor samples and 32 normal samples. Log2 transformation was performed for FPKM formatted RNA-seq data. The mRNA expression levels of unpaired samples and paired samples were visualized by the ggplot2 package. The ROC curve was drawn using the pROC package.

### Establishment of a *CBXs*-related gene model

A Cox regression analysis was performed to examine the prognostic significance of *CBXs*. Significantly prognostic *CBXs* were selected for additional analysis. Based on these prognostic *CBX*, a prognostic model was constructed using LASSO Cox regression analysis. Based on the median risk score, gastric cancer patients were subdivided into high-risk and low-risk groups. Kaplan-Meier analysis was performed to compare the overall survival time of the two subgroups. ROC (time receiver-operating characteristic) analysis was performed to examine the predictive accuracy of each gene and risk score. For model validation, the GSE84437 [32] dataset (*n* = 433) of gastric cancer was acquired from the GEO database (https://www.ncbi.nlm.nih.gov/geo/). Spearman’s correlation between risk score and immune infiltration was analyzed using the McP-counter algorithm. A predicted nomogram was developed to predict the overall survival of gastric cancer patients at 1, 2, and 3 years. A forest plot was used to reveal the *P*-value, HR, and 95% CI of each variable through the “forestplot” R package.

### Correlation analysis of risk score and immunotherapy

The correlation of model risk scores with immune checkpoints and TMB was analyzed in the TCGA-STAD and GSE84437 datasets by the ggpubr package. The TIDE and T cell dysfunction scores of TCGA-STAD tumor samples were predicted from the TIDE database (http://tide.dfci.harvard.edu/login/) and the levels of these scores were compared between high and low risk groups. TCGA-STAD IPS and MIS scores data were downloaded from the TCIA database (https://tcia.at/home) to compare the levels of these scores for high and low risk groups.

### TISIDB analysis

The TISIDB database (http://cis.hku.hk/TISIDB) merges 988 reported immune-related genes in the tumor microenvironment and provides a relationship between genes and immune cell infiltration by analyzing high-throughput screening data and genomics, transcriptomics, as well as clinical data [33]. Here, we established the relationships among expression levels of *CBXs*, clinical information, and subtype, and evaluated the correlations between *CBXs* expression and lymphocytes in gastric cancer.

### GEPIA database

GEPIA (http://gepia.cancer-pku.cn/) is a web server with RNA sequencing expression data from the TCGA and GTEx projects [34]. Transcriptional expression differences of *CBXs* were compared between gastric cancer and normal gastric tissues.

### Human protein atlas

The Human Protein Atlas (https://www.proteinatlas.org) is a website comprising immunohistochemistry-based expression cell lines and tissue data for most identified genes [35]. We directly compared protein expression levels of different *CBXs* family members by obtaining immunohistochemical images between human normal and gastric cancer tissues.

### Kaplan-Meier plotter database

The predictive values of *CBXs* in gastric cancers were analyzed by the Kaplan-Meier plotter (http://kmplot.com/analysis/) [36]. Differences with *P*-values less than 0.05 (*P* < 0.05) were considered statistically significant.

### GeneMANIA

GeneMANIA (http://www.genemania.org) is a flexible website that provides gene functions, protein interactions, relationships of genes and datasets, functionally similar genes, as well as similar genes with shared properties [37].

### Metascape

Metascape (http://metascape.org) is a predictable and instinctive tool for gene annotation and gene enrichment analysis [38]. GO and KEGG in Metascape were used to analyze the functions of *CBXs* and *CBXs* co-expression genes.

### TIMER

TIMER (https://cistrome.shinyapps.io/timer/) is a detailed resource for the systematic analysis of immune infiltrates [39, 40]. The correlation between the expression of *CBXs* and the abundance of immune cell infiltration was analyzed in the “Gene” module. Clinical relevance of infiltrated immune cells and *CBXs* expression in a multivariable Cox proportional hazards model were evaluated in the “Survival” module.

### Enrichr

Enrichr (http://amp.pharm.mssm.edu/Enrichr/) is a comprehensive online resource for curated gene sets and gene function analysis [41]. Enrichr contains 184 annotated gene sets from 102 gene set libraries for analysis and download, including transcription, pathways, ontologies, diseases/drugs, cell types, etc.

### Statistical analysis

The online databases were used to automatically perform statistical analyses, and the part of the code analysis was completed using the R package. For categorical variables, the chi-squared test was used, but for continuous variables, the Wilcoxon signed-rank test was applied. For comparisons, Spearman’s correlation analysis was utilized. *P* < 0.05 was considered statistically significant.

### Data availability

The data supporting our results of this work are obtainable from TCGA (https://portal.gdc.cancer.gov/) and other data in the paper can be obtained from the corresponding author based on reasonable request.

## Supplementary Materials

Supplementary Figures

Supplementary Tables
