# Supplementary material for: CBXs-related prognostic gene signature correlates with immune microenvironment in gastric cancer
Source: Aging (Albany NY). 2022 Aug 14;14(15):6227–54. doi: 10.18632/aging.204214 (PMC9417237; doi:10.18632/aging.204214)
Supplement: Supplementary Figures [file aging-14-204214-s001.pdf]

## SUPPLEMENTARY FIGURES

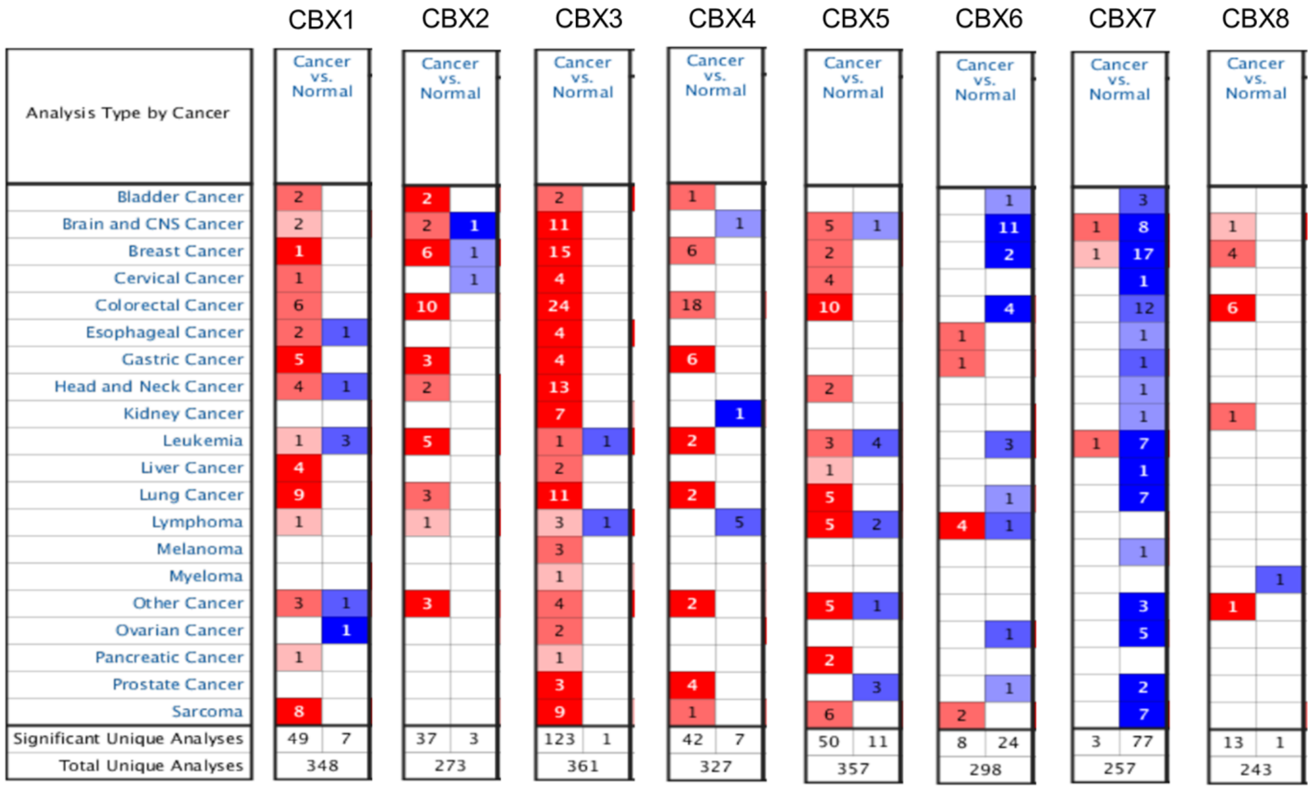


**Supplementary Figure 1. Transcriptional expression comparison of *CBXs* in 20 different types of cancer diseases analyzed by ONCOMINE database.** mRNA level was compared by student's *t*-test. Threshold setting: *P*-value <0.01; fold change: 1.5; gene rank: 10%.

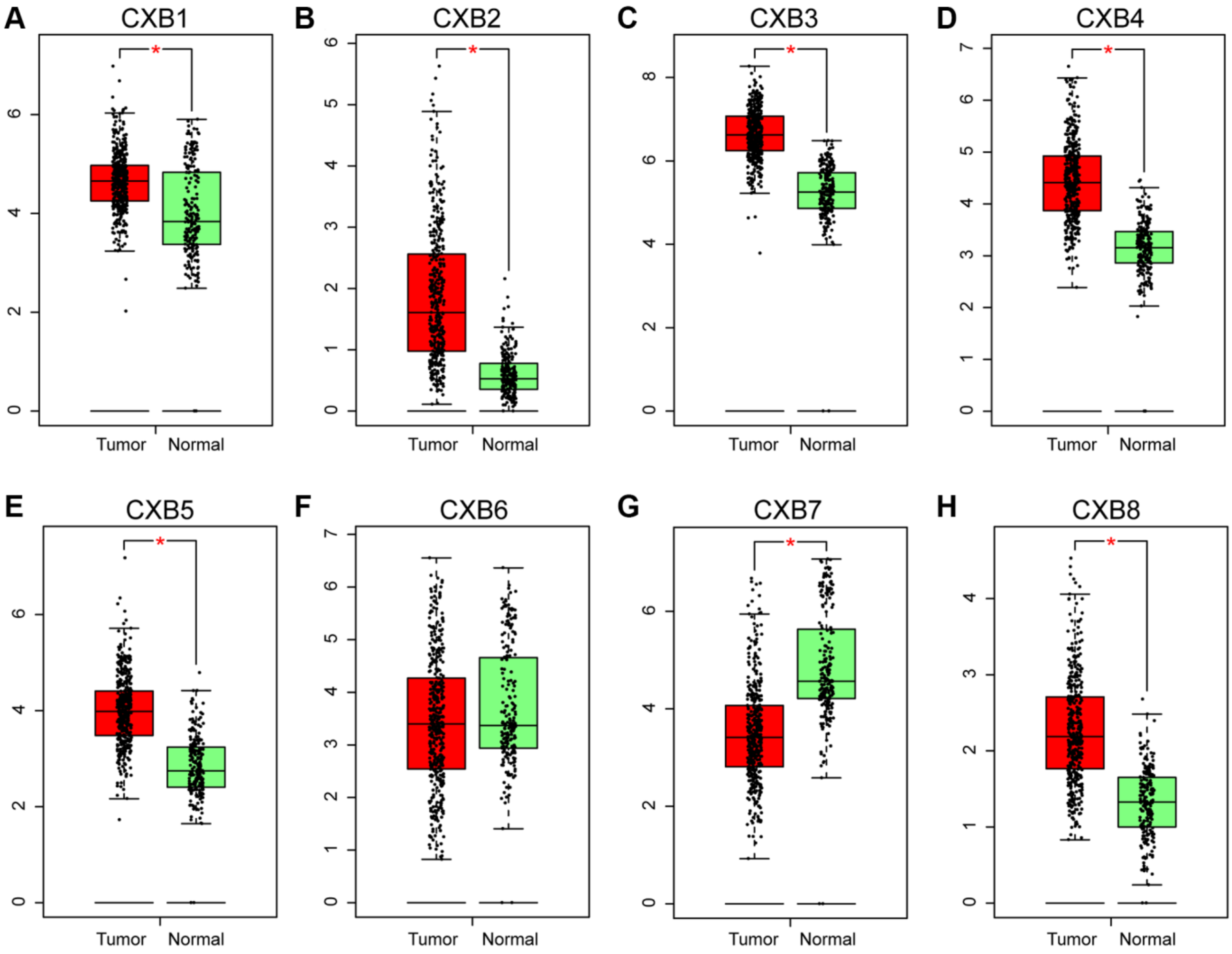


**Supplementary Figure 2. Comparison of the mRNA expression level of 8 *CBXs* family members in gastric cancer tissues and adjacent normal tissues analyzed by the GEPIA online database.** (**A**) *CBX1*; (**B**) *CBX2*; (**C**) *CBX3*; (**D**) *CBX4*; (**E**) *CBX5*; (**F**) *CBX6*; (**G**) *CBX7*; (**H**) *CBX8*. $^{*}P < 0.05$.

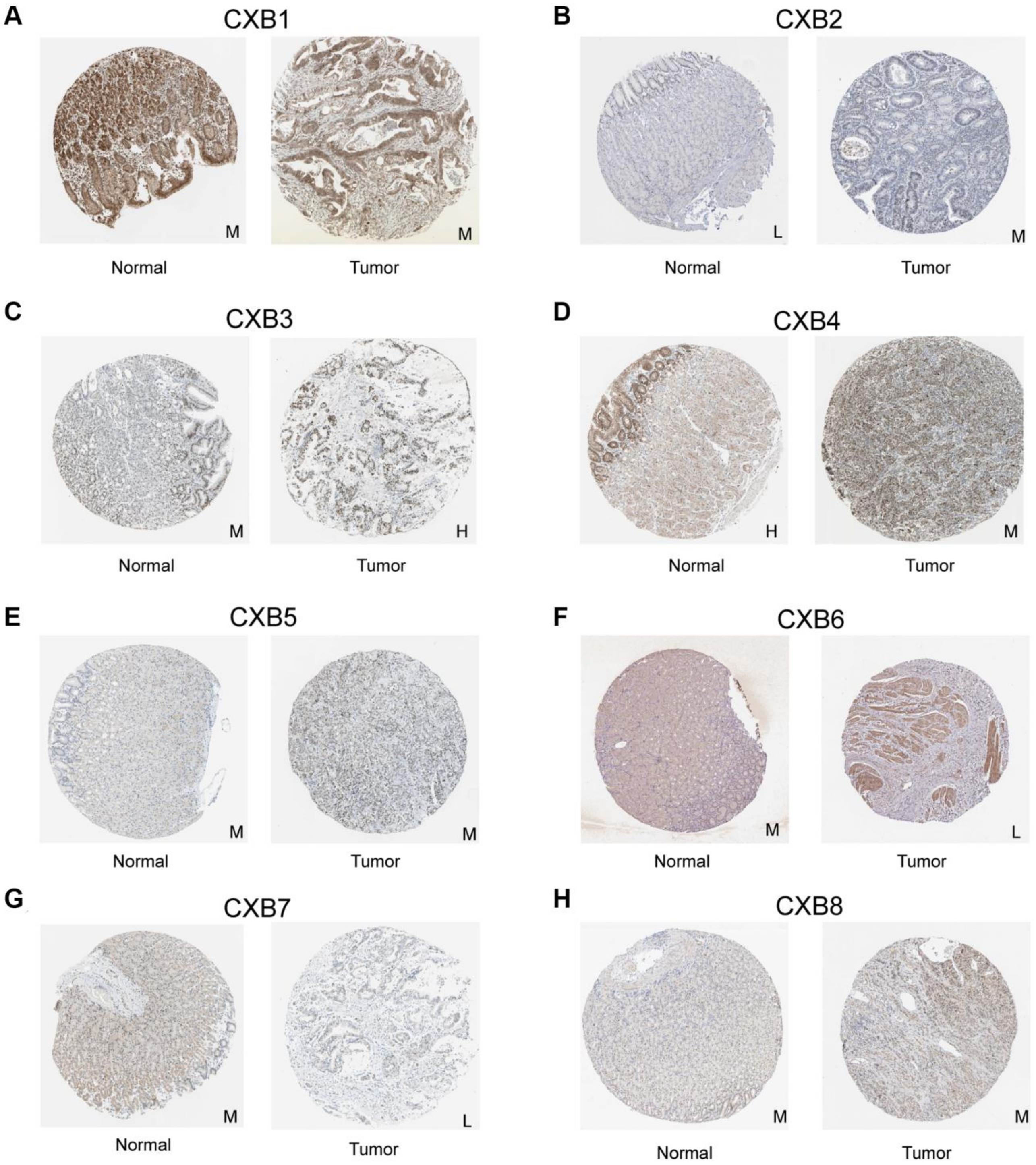


**Supplementary Figure 3. Representative immunohistochemistry images of *CBXs* family members in gastric cancer tissues and normal tissues using the human protein atlas database.** (**A**) *CBX1*; (**B**) *CBX2*; (**C**) *CBX3*; (**D**) *CBX4*; (**E**) *CBX5*; (**F**) *CBX6*; (**G**) *CBX7*; (**H**) *CBX8*. Abbreviations: L: low expression; M: middle expression; H: high expression.

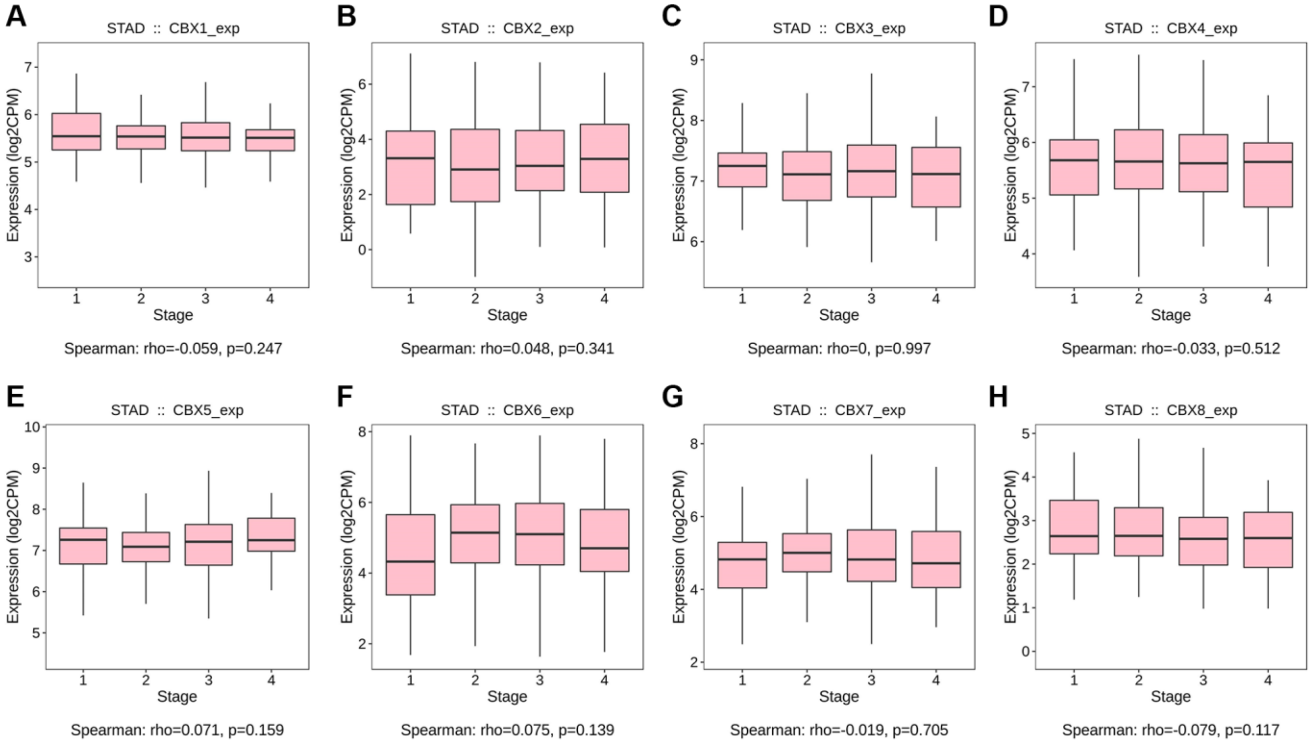


**Supplementary Figure 4. Spearman's correlation analysis of the *CBXs* expression and clinical stages in gastric cancer via the TISIDB database.** (**A**) *CBX1*; (**B**) *CBX2*; (**C**) *CBX3*; (**D**) *CBX4*; (**E**) *CBX5*; (**F**) *CBX6*; (**G**) *CBX7*; (**H**) *CBX8*.

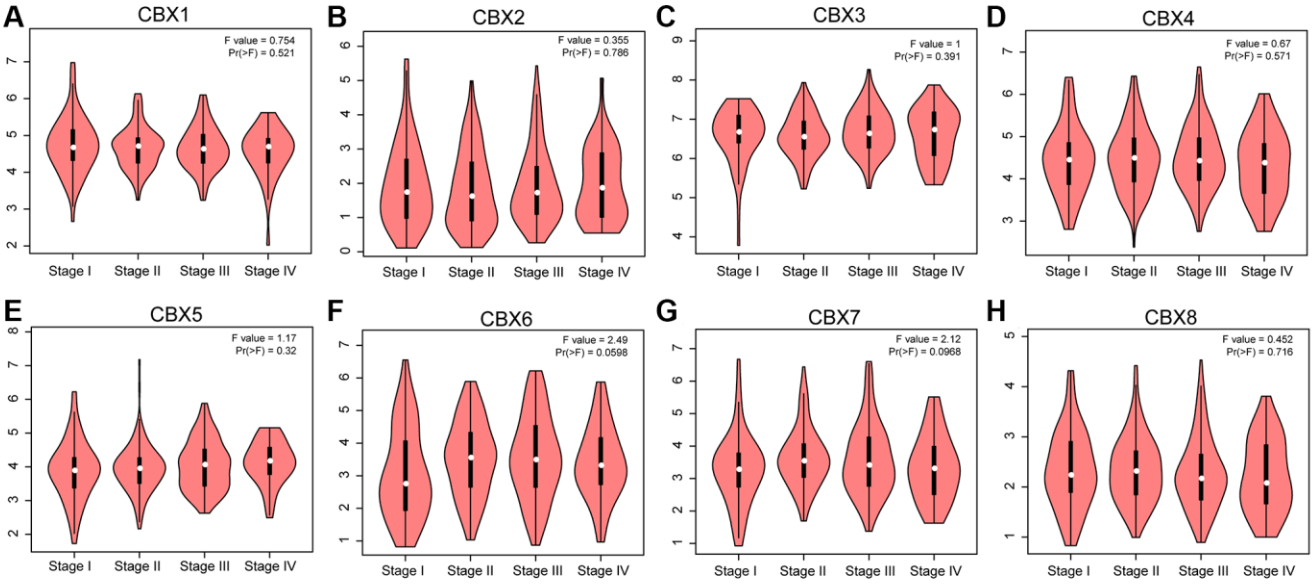


**Supplementary Figure 5. Correlation between mRNA expression of *CBXs* family members and pathological stages of gastric cancer patients analyzed by GEPIA.** (**A**) *CBX1*; (**B**) *CBX2*; (**C**) *CBX3*; (**D**) *CBX4*; (**E**) *CBX5*; (**F**) *CBX6*; (**G**) *CBX7*; (**H**) *CBX8*.

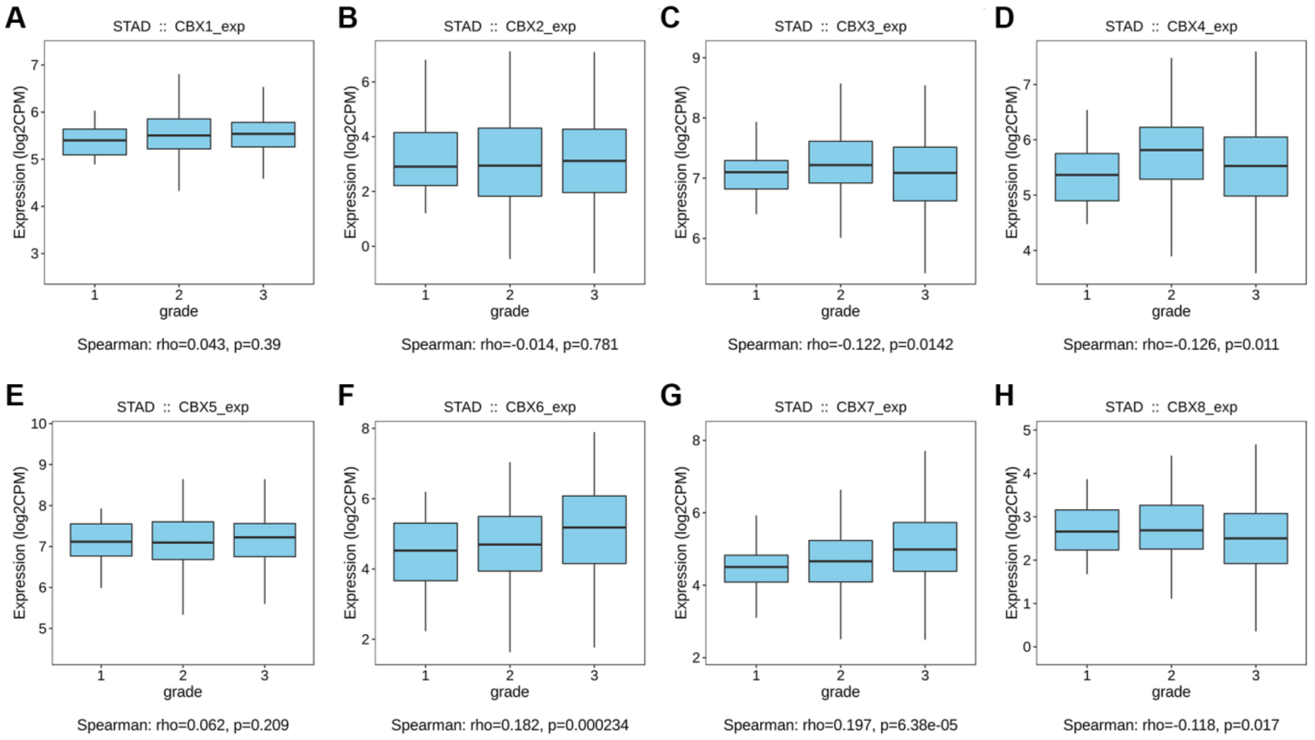


**Supplementary Figure 6. Spearman's correlation analysis of the *CBXs* expression and clinical grades in gastric cancer using the TISIDB database.** (**A**) *CBX1*; (**B**) *CBX2*; (**C**) *CBX3*; (**D**) *CBX4*; (**E**) *CBX5*; (**F**) *CBX6*; (**G**) *CBX7*; (**H**) *CBX8*.

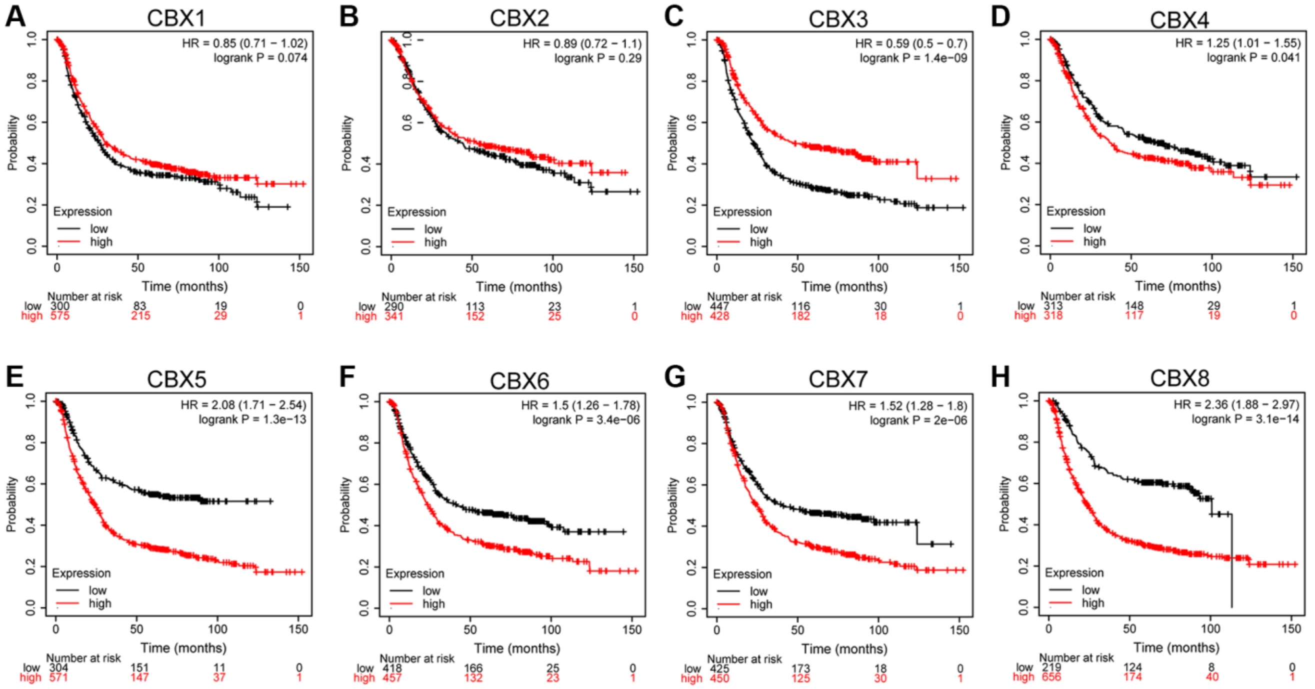


**Supplementary Figure 7. The overall survival rate of *CBXs* family members in gastric cancer patients using the microarray data.** (**A**) *CBX1*; (**B**) *CBX2*; (**C**) *CBX3*; (**D**) *CBX4*; (**E**) *CBX5*; (**F**) *CBX6*; (**G**) *CBX7*; (**H**) *CBX8*.

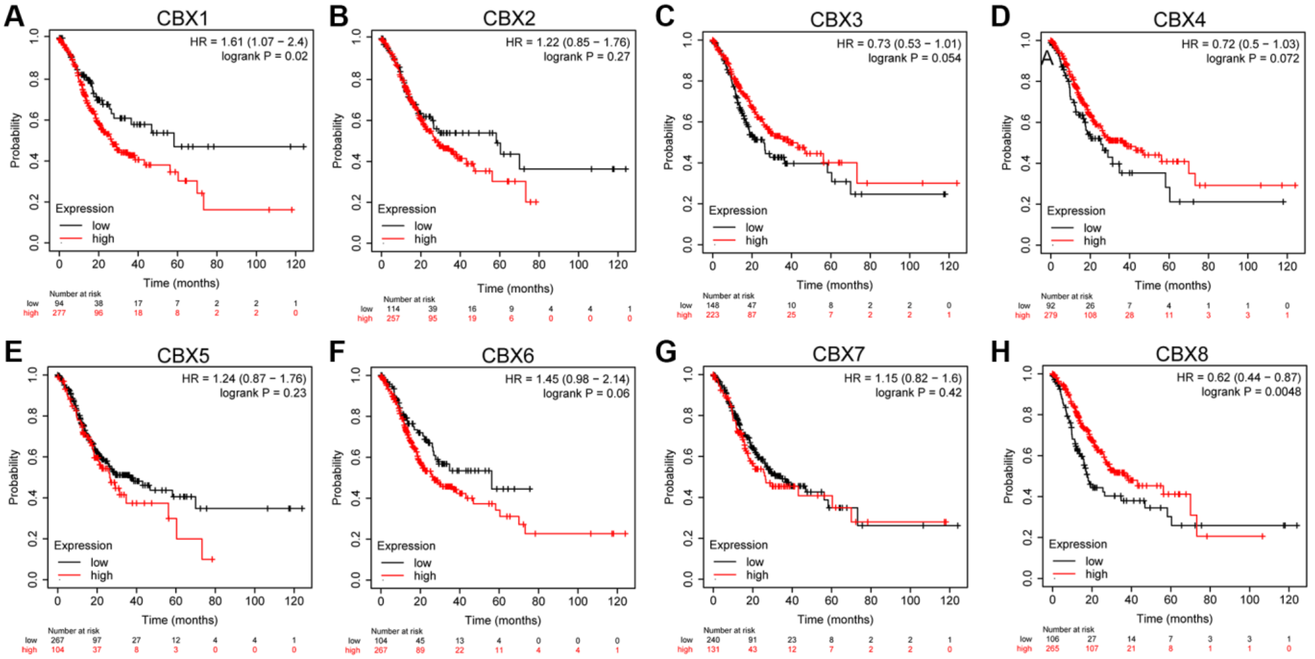


**Supplementary Figure 8. The overall survival rate of *CBXs* in gastric cancer was analyzed in Kaplan–Meier plotter.** (**A**) *CBX1*; (**B**) *CBX2*; (**C**) *CBX3*; (**D**) *CBX4*; (**E**) *CBX5*; (**F**) *CBX6*; (**G**) *CBX7*; (**H**) *CBX8*. The *P* values were calculated using the log-rank test.

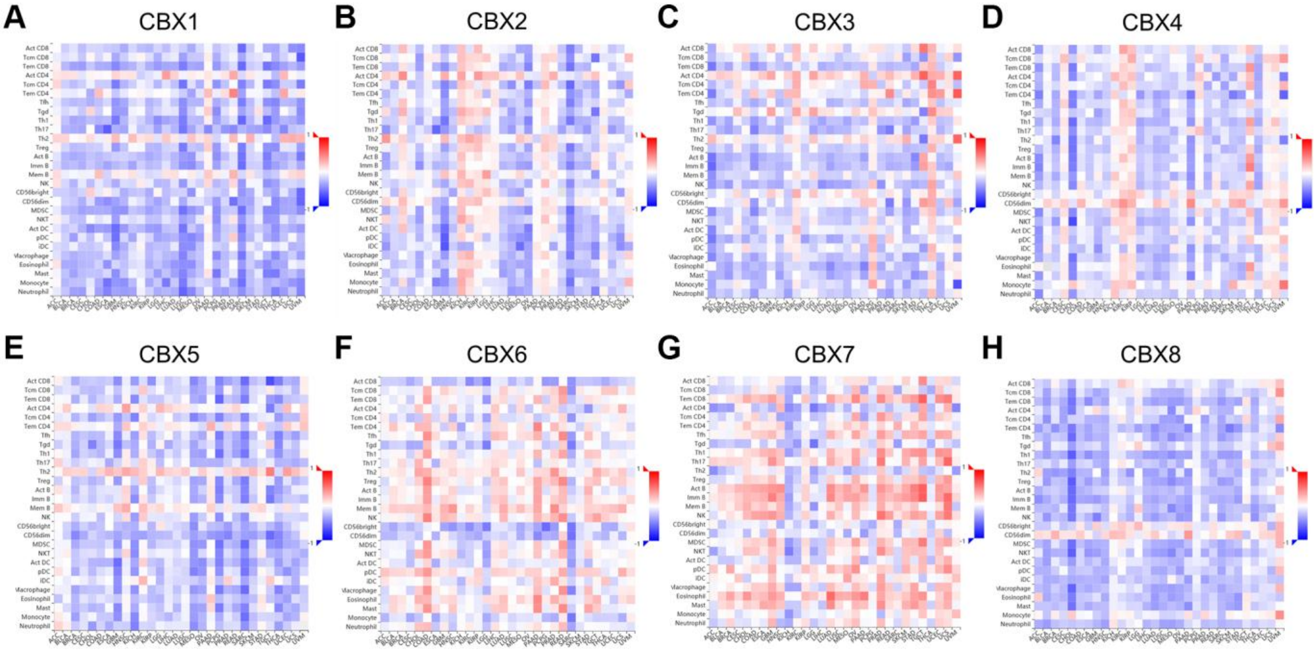


**Supplementary Figure 9. Spearman's correlation of *CBXs* expression with the abundance of tumor-infiltrating lymphocytes (TILs) was analyzed in various cancers by TISIDB.** (**A**) *CBX1*; (**B**) *CBX2*; (**C**) *CBX3*; (**D**) *CBX4*; (**E**) *CBX5*; (**F**) *CBX6*; (**G**) *CBX7*; (**H**) *CBX8*. The red color indicated the positive correlation, and the blue color indicated the negative correlation.

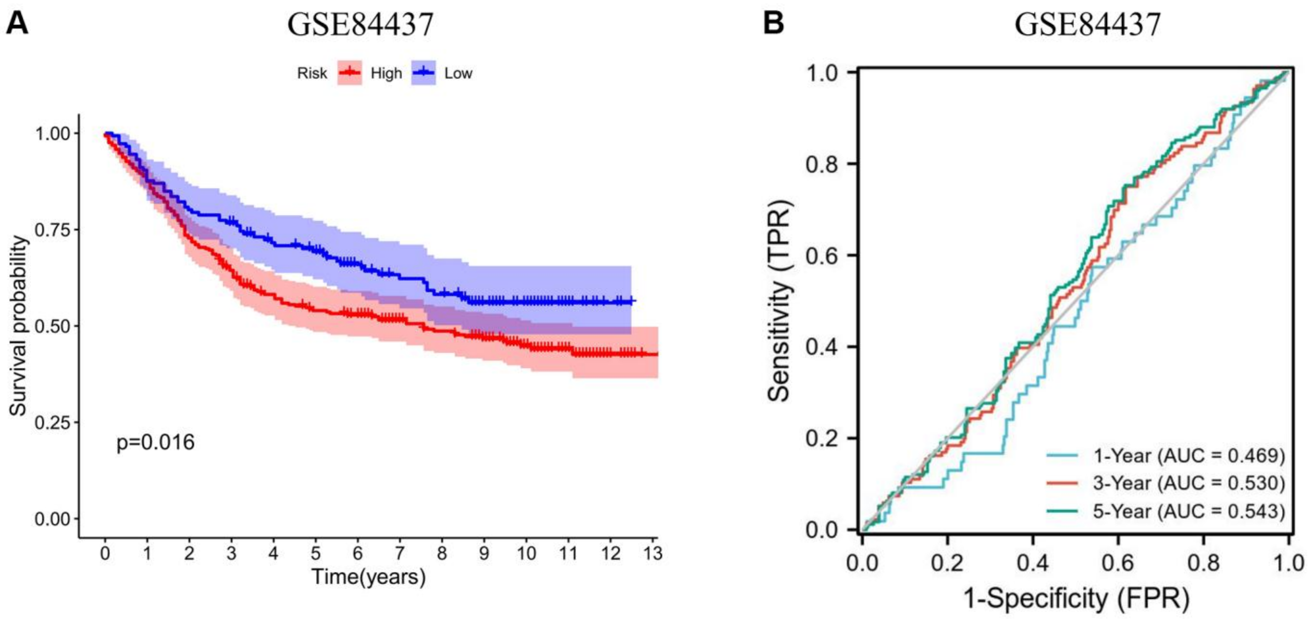


**Supplementary Figure 10. Validation of the CBXs prognostic model in GSE84437.** (**A**) Overall survival curves for gastric cancer patients in the high/low risk group. (**B**) The ROC curve of measuring the predictive value. Abbreviation: CBXs, chromobox proteins.

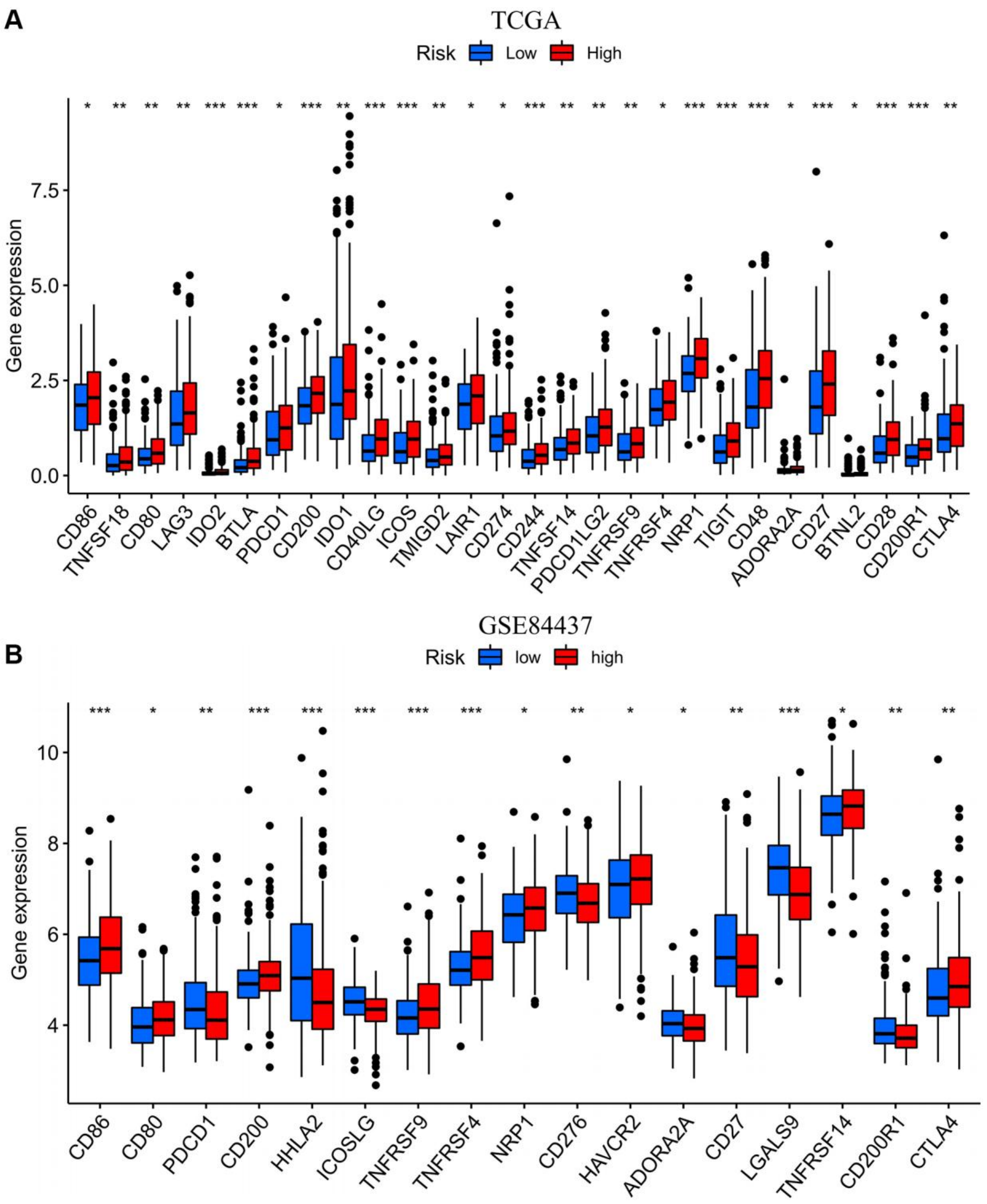


**Supplementary Figure 11. Correlation analysis between risk score and immune checkpoints.** Wilcox. test statistical analysis found significant differences in immune checkpoints between low and high risk score patients in TCGA-STAD (**A**) and GSE84437 (**B**) data sets. $^{*}p < 0.05$; $^{**}p < 0.01$; $^{***}p < 0.001$.
