# Supplementary material for: CBXs-related prognostic gene signature correlates with immune microenvironment in gastric cancer
Source: Aging (Albany NY). 2022 Aug 14;14(15):6227–54. doi: 10.18632/aging.204214 (PMC9417237; doi:10.18632/aging.204214)
Supplement: Supplementary Tables [file aging-14-204214-s002.pdf]

SUPPLEMENTARY TABLES

Supplementary Table 1. Significant changes of the mRNA levels of CBXs between gastric cancer and normal gastric tissues were analyzed by ONCOMINE.

Gene	Type	Fold change	*P*-value	References
CBX1	Gastric Adenocarcinoma	2.415	4.52E-06	[2]
	Diffuse Gastric Adenocarcinoma	1.516	2.83E-08	[1]
	Gastric Mixed Adenocarcinoma	1.66	2.25E-06	[1]
	Gastric Intestinal Type Adenocarcinoma	1.613	2.38E-13	[1]
	Gastric Intestinal Type Adenocarcinoma	2.116	2.21E-13	[3]
CBX2	Diffuse Gastric Adenocarcinoma	2.29	6.01E-09	[2]
	Gastric Mixed Adenocarcinoma	2.077	3.75E-04	[2]
	Gastric Intestinal Type Adenocarcinoma	4.485	1.70E-09	[3]
CBX3	Gastric Intestinal Type Adenocarcinoma	3.014	6.64E-14	[3]
CBX4	Gastric Intestinal Type Adenocarcinoma	1.783	2.55E-17	[1]
	Gastric Mixed Adenocarcinoma	1.955	3.03E-06	[1]
	Diffuse Gastric Adenocarcinoma	1.73	4.23E-04	[1]
	Diffuse Gastric Adenocarcinoma	2.466	2.45E-05	[3]
	Gastric Mixed Adenocarcinoma	3.314	2.29E-06	[3]
	Gastric Mixed Adenocarcinoma	1.625	7.18E-04	[2]
CBX6	Diffuse Gastric Adenocarcinoma	1.758	8.38E-05	[1]
CBX7	Diffuse Gastric Adenocarcinoma	−1.656	9.09E-05	[2]

Abbreviation: CBXs: Chromobox proteins.

REFERENCES

1. Chen X, Leung SY, Yuen ST, Chu KM, Ji J, Li R, Chan AS, Law S, Troyanskaya OG, Wong J, So S, Botstein D, Brown PO. Variation in gene expression patterns in human gastric cancers. Mol Biol Cell. 2003; 14:3208–15. https://doi.org/10.1091/mbc.e02-12-0833 PMID:12925757

2. Cho JY, Lim JY, Cheong JH, Park YY, Yoon SL, Kim SM, Kim SB, Kim H, Hong SW, Park YN, Noh SH, Park ES, Chu IS, et al. Gene expression signature-based prognostic risk score in gastric cancer. Clin Cancer Res. 2011; 17:1850–7. https://doi.org/10.1158/1078-0432.CCR-10-2180 PMID:21447720

3. D'Errico M, de Rinaldis E, Blasi MF, Viti V, Falchetti M, Calcagnile A, Sera F, Saieva C, Ottini L, Palli D, Palombo F, Giuliani A, Dogliotti E. Genome-wide expression profile of sporadic gastric cancers with microsatellite instability. Eur J Cancer. 2009; 45:461–9. https://doi.org/10.1016/j.ejca.2008.10.032 PMID:19081245

Supplementary Table 2. Drug targets of CBXs in gastric cancer (Enrichr).

Term	Overlap	*P*-value	Odds ratio	Combined score	Genes
Prednisolone-184 mg/kg in Water-Rat-Heart-5d-up	3/257	0.005418	8.337966	43.50722	CBX6
Phenacetin-619 mg/kg in Corn Oil-Rat-Kidney-5d-up	3/260	0.005595	8.241758	42.74019	CBX6
Pramoxine-526 mg/kg in Corn Oil-Rat-Heart-1d-up	3/261	0.005655	8.210181	42.48917	CBX6
44'-Methylenedianiline-81 mg/kg in Corn Oil-Rat-Kidney-5d-up	3/261	0.005655	8.210181	42.48917	CBX6
Bromisovalum-250 mg/kg in Corn Oil-Rat-Kidney-5d-up	3/267	0.006022	8.025682	41.02991	CBX6
6-Mercaptopurine-25 mg/kg in Corn Oil-Rat-Kidney-5d-up	3/269	0.006147	7.966012	40.56069	CBX6
Bromisovalum-250 mg/kg in Corn Oil-Rat-Kidney-1d-up	3/272	0.006338	7.878151	39.87225	CBX6
2-Acetylaminofluorene-30 mg/kg in CMC-Rat-Kidney-3d-up	3/273	0.006403	7.849294	39.64677	CBX6
123-Trichloropropane-108 mg/kg in CMC-Rat-Kidney-1d-up	3/274	0.006468	7.820647	39.42326	CBX6
Ascorbic Acid-2000 mg/kg in Water-Rat-Kidney-1d-up	3/279	0.006798	7.680492	38.33433	CBX6

Abbreviation: CBXs: Chromobox proteins.

Supplementary Table 3. microRNA targets of CBXs in gastric cancer (Enrichr).

Term	Overlap	*P*-value	Odds ratio	Combined score	Genes
mmu-miR-493	10/1997	2.33E-04	3.576794	29.91359	CBX2/5
hsa-miR-1296	8/1377	4.43E-04	4.149808	32.04482	CBX2/5/6
mmu-miR-5128	7/1108	6.66E-04	4.512635	33.00666	CBX1/2/4/7
hsa-miR-2277-3p	9/1993	0.001132	3.225575	21.88069	CBX3/4/7
mmu-miR-293	4/382	0.001866	7.479432	47.00003	CBX6/7
mmu-miR-2183	7/1335	0.001985	3.745318	23.30452	CBX3/5/6
mmu-miR-3099	7/1572	0.004978	3.180662	16.86609	CBX6/7
hsa-miR-4730	4/516	0.005485	5.537099	28.82443	CBX2/4/5
hsa-miR-566	5/877	0.006797	4.072324	20.32605	CBX2/5/7
mmu-miR-1306-5p	7/1681	0.007174	2.97442	14.68542	CBX7

Abbreviation: CBXs: Chromobox proteins.

Supplementary Table 4. Transcriptional factor targets of CBXs in gastric cancer (Enrichr).

Term	Overlap	*P*-value	Odds ratio	Combined score	Genes
TEAD4 (human)	10/1354	8.31E-06	5.275375	61.71111	CBX2/6/7/8
NRF1 (human)	9/1356	6.26E-05	4.740834	45.88685	CBX1/2/5/7
HINFP (human)	13/3047	9.07E-05	3.047494	28.36607	CBX2/3/7
WT1 (human)	12/2689	1.28E-04	3.18759	28.58052	CBX2/3/6/7/8
E2F1 (human)	15/4207	1.52E-04	2.546776	22.39494	CBX1/2/3/4/5/6/7/8
EGR1 (mouse)	8/1617	0.001284	3.533881	23.52762	CBX2/3/7
SP3 (human)	7/1332	0.001959	3.753754	23.40551	CBX2/4/7/8
PCBP1 (human)	7/1360	0.002207	3.676471	22.48527	CBX2/4/7/8
TCFAP2A (human)	7/1367	0.002273	3.657644	22.26283	CBX2/4/8

Abbreviation: CBXs: Chromobox proteins.
